# Community health workers at the dawn of a new era: 11. CHWs leading the way to “Health for All”

**DOI:** 10.1186/s12961-021-00755-5

**Published:** 2021-10-12

**Authors:** Henry B. Perry, Mushtaque Chowdhury, Miriam Were, Karen LeBan, Lauren Crigler, Simon Lewin, David Musoke, Maryse Kok, Kerry Scott, Madeleine Ballard, Steve Hodgins

**Affiliations:** 1 Department of International Health, Johns Hopkins Bloomberg School of Public Health, Baltimore, MD USA; 2 BRAC University, Dhaka, Bangladesh; 3 UZIMA Foundation, Nairobi, Kenya; 4 Independent Consultant, Washington, DC USA; 5 Crigler Consulting, LLC, Hillsborough, NC USA; 6 Norwegian Institute of Public Health, Oslo, Norway and Health Systems Research Unit, South African Medical Research Council, Cape Town, South Africa; 7 Department of Disease Control and Environmental Health, School of Public Health, College of Health Sciences, Makerere University, Kampala, Uganda; 8 Department of Global Health, KIT Royal Tropical Institute, Amsterdam, The Netherlands; 9 Independent Consultant, Toronto, Canada; 10 Community Health Impact Coalition, New York, NY USA; 11 Department of Health System Design and Global Health, Icahn School of Medicine at Mount Sinai, New York City, NY USA; 12 School of Public Health, University of Alberta, Edmonton, AB Canada

**Keywords:** Community health workers, Community health worker programmes, Lay health workers, Community-based primary healthcare, Primary healthcare, Sustainable development goals, Ending preventable child and maternal deaths, Universal Health Coverage

## Abstract

**Background:**

This is the concluding paper of our 11-paper supplement, “Community health workers at the dawn of a new era”.

**Methods:**

We relied on our collective experience, an extensive body of literature about community health workers (CHWs), and the other papers in this supplement to identify the most pressing challenges facing CHW programmes and approaches for strengthening CHW programmes.

**Results:**

CHWs are increasingly being recognized as a critical resource for achieving national and global health goals. These goals include achieving the health-related Sustainable Development Goals of Universal Health Coverage, ending preventable child and maternal deaths, and making a major contribution to the control of HIV, tuberculosis, malaria, and noncommunicable diseases. CHWs can also play a critical role in responding to current and future pandemics. For these reasons, we argue that CHWs are now at the dawn of a new era. While CHW programmes have long been an underfunded afterthought, they are now front and centre as the emerging foundation of health systems. Despite this increased attention, CHW programmes continue to face the same pressing challenges: inadequate financing, lack of supplies and commodities, low compensation of CHWs, and inadequate supervision. We outline approaches for strengthening CHW programmes, arguing that their enormous potential will only be realized when investment and health system support matches rhetoric. Rigorous monitoring, evaluation, and implementation research are also needed to enable CHW programmes to continuously improve their quality and effectiveness.

**Conclusion:**

A marked increase in sustainable funding for CHW programmes is needed, and this will require increased domestic political support for prioritizing CHW programmes as economies grow and additional health-related funding becomes available. The paradigm shift called for here will be an important step in accelerating progress in achieving current global health goals and in reaching the goal of Health for All.

Key message box 1: Summary **Key findings**

- National community health worker (CHW) programmes face many challenges which prevent them from reaching their full potential.
- The lack of national political support and sustainable funding (from both domestic and donor sources) for national CHW programmes is perhaps the most critical challenge facing these programmes.
- Other key challenges these programmes face include provision of programme supplies and commodities, compensation of CHWs, and supervision.

**Key implications**

- Developing, expanding, and strengthening national CHW programmes represent promising approaches to accelerating progress in achieving the global health goals of Universal Health Coverage, ending preventable child and maternal deaths, and achieving “Health for All”.
- By building on current evidence and best practices, and investing in rigorous monitoring, evaluation, and implementation research, national CHW programmes have the potential to lead the way in reaching “Health for All”.

## Background

This is the 11th and final paper in our series, “Community health workers at the dawn of a new era”. The first paper provides an introduction to the current status of national CHW programmes, their role within the peripheral health system and the community health system, and some of the long-standing tensions related to the identity and orientation of CHWs in national health systems [1]. The second and third papers address coordination, planning, partnerships, and governance—all critical issues for national CHW programmes, which intersect with so many components of society and government and are themselves complex entities [2, 3]. As these programmes become stronger and more embedded within health systems rather than, as in many countries, stand-alone projects mostly funded by external donors, coordination, planning, partnerships, and governance gradually take on increasing importance. Long-term financing of CHW programmes, as discussed in the fourth paper [4], is a critically important issue as national CHW programmes gain momentum. Making a compelling case based on the expected return on investment both for improving population health and for promoting socioeconomic development will be critical for generating the political will to ensure stronger long-term domestic funding for these programmes as well as more immediate donor funding and longer-term support from donors as well.

The fifth paper addresses roles and tasks that CHWs perform—an increasingly complex issue as the evidence grows regarding the range of functions that trained and supported CHWs are capable of fulfilling [5]. There is a steady but persistently increasing professionalization of CHW roles. There is also an emerging dual cadre of CHWs in which professionalized CHWs are supervising lower tiers of CHWs, who are often volunteers responsible for a small number of households. These dynamics along with the changing priorities of countries as they pass through the epidemiologic transition means that the roles and tasks of CHWs will continue to evolve, as the experience with the national CHW programme in Brazil demonstrates.

The training of CHWs [6] (the sixth paper) along with their supervision [7] (the seventh paper) and incentives/reimbursement [8] (the eighth paper) are all elements of national CHW programmes that influence programme performance. New approaches that more effectively meet the needs of CHWs are being tried in many programmes. There is growing recognition of the need for fair remuneration for full- and part-time CHWs. A role also remains for voluntary CHWs, who can make important contributions as long as they are not be expected to work more than a few hours per week or perform the role of regular full- or part-time workers.

The need for national CHW programmes to be embedded in both communities and health systems is highlighted in the ninth paper [9]. The tenth paper [10] focuses on the performance of CHWs and CHW programmes and approaches to assessing performance. A comprehensive approach is needed since the effectiveness of the whole programme is dependent on how well each component of the system is functioning; addressing one component without consideration of the other components is not likely to improve programme effectiveness. The paper highlights the importance of both human and technical components of performance. Table 1 contains the key messages and key implications from each of these 10 papers.

This series of papers builds on previous work of our writing team, most notably the widely used 2014 CHW Reference Guide, which was intended to guide CHW programme managers and policy-makers in developing and strengthening CHW programmes at scale [11]. In addition, we draw heavily on recently released World Health Organization (WHO) guidelines guidelines for CHW programmes [12] and a compendium of case studies of 29 national CHW programmes [13].

**Table 1:** Key findings and key implications of the first 10 papers in the supplement on CHWs at the dawn of a new era

Title of paper	Key findings	Key implications	References
*Overview and Tensions Confronting Large-Scale Programmes*	Across different settings, CHWs play diverse roles; in large-scale programmes they have functions related to both health education and helping extend or bridge to primary healthcare services CHWs fall along a spectrum from lay/volunteer to more professionalized Nongovernmental organizations have played an important role in the development of CHW programmes and continue to engage with government in many public-sector CHW programmes While evidence-informed models, interventions, and tools are important, for programmes to be effective, the approaches used also need to be responsive to the local context Over the past several decades, CHWs have played important roles in vertically delivered disease control programmes; increasingly, they are taking on broader roles in more integrated primary healthcare services	The CHW is one actor in a complex, dynamic, primary healthcare system comprised of diverse interacting actors, each having agency, interests, perspectives, and values Robust delivery of services at the most peripheral level of the primary healthcare system—reaching all who could benefit—requires functional systems enabling CHWs to play a constructive part, and that, in turn, depends on their role being well understood and appropriately supported	[1]
*Planning, Coordination, and Partnerships*	CHW programmes in many countries have been shown to be effective in improving health. However, they are often hindered by weak health systems Poor planning, lack of coordination, and failed partnerships have produced lacklustre CHW programmes in countries The development, strengthening, and governance of CHW programmes need to take into account the country’s health system’s socioeconomic and political context and tailor solutions to the country’s political economy	The key principles on which there is broad agreement in making CHW programmes effective include strengthening planning, coordination, and partnerships	[2]
*Governance*	This paper discusses the following important questions that decision-makers need to consider in relation to governing CHW programmes: How and where within political structures are policies made for CHW programmes? Who implements decisions regarding CHW programmes, and at what levels of government? What laws and regulations are needed to support the programme? How should the programme be adapted across different settings or groups within the country or region?	Governing CHW programmes is complex because of the location of these programmes at the interface between the formal health system and communities How CHW programmes are governed affects many other processes in these programmes, including management, resourcing, accountability, and ultimately performance and sustainability The most appropriate and acceptable models for governing CHW programmes depend on communities, on local health systems, and on the political system in which the programme is located. Stakeholders in each setting need to consider what systems are currently in place and how they might be adapted to and aligned with local needs and systems The development or revitalization of large-scale CHW programmes in a number of contexts provides opportunities to explore how different models of governing CHW programmes impact on the quality of care delivered, their responsiveness to local needs, and their sustainability	[3]
*Financing*	CHWs can be a cost-effective way to extend health services to hardest-to-reach communities Investing in CHWs can lead to short-term and long-term cost savings in the health system and help achieve broader societal goals such as women and youth empowerment Investing in community health yields a 10:1 return on investment Despite compelling evidence on their effectiveness, CHW programmes are inadequately funded The lack of national political support and domestic funding for national CHW programmes is perhaps the most critical challenge facing these programmes. Sixty percent of funding for CHW programmes in sub-Saharan Africa is from donors, and most of this is for vertical disease-specific programmes Determining CHW programme costs and funding requirements is critical for strengthening and expanding these programmes Mobilizing political will is a prerequisite for moving forward with stronger financing for CHW programmes	Secure, long-term financing of CHW programmes is critically important for community health programmes to reach their full potential Making a compelling case based on the expected return on investment both for improving population health and for promoting socioeconomic development will be critical for generating the political will to ensure long-term domestic funding for CHW programmes Countries need to be proactive in obtaining alternative, sustainable financing for CHW programmes in addition to the existing traditional funding from donors and domestic resources	[4]
*Roles and tasks*	This article provides a list of 10 questions that can help programme planners think about important issues when determining CHW roles and tasks: How effective and safe will it be to use CHWs to perform a specific task? Are CHWs’ roles and tasks likely to be regarded as acceptable by CHWs and their clients and communities? Are CHWs’ roles and tasks considered relevant by the community? Is there a good match between the CHWs and the roles and tasks expected of them? How many tasks and activities should each CHW take on? What is most feasible for the health system? When and where will CHWs deliver each task and how much workload will it require? What kind of skills and training will the CHW need when performing specific tasks? What type of health system support will the CHW require when performing the task? How much will it cost to use CHWs to perform the task?	When planning new CHW roles or expanding the roles of existing CHWs, programme planners need to base their decisions on global and local evidence and guidance Planners need to consider the effectiveness and safety of relevant tasks performed by CHWs They also need to assess whether the recommended CHW roles and tasks are considered acceptable and appropriate by their target population and by the CHWs themselves and those who support them Finally, planners need to think about the practical and organizational implications of each task for their particular setting with regard to training requirements, health systems support, work location, workload, and programme costs	[5]
*Recruitment, training and continuing education*	Training is a comprehensive and dynamic (meaning not static and in need of continuous modification) element of CHW programmes that needs to be well funded and professionalized (meaning provided with a high level of skill and expertise) Training must be seen as much more than just pre-service training, but rather as ongoing iterative training Training for effective CHW programmes also needs to be seen not only as training of CHWs, but also training the community, supervisors of CHWs, and others within the health system in order to help these stakeholders appreciate, understand, and make effective use of CHWs	Professionalism of CHW training means ongoing assessment of quality and continuous quality improvement, which will require programmes to have adequate capacity and resources for these activities CHW roles will continue to change over time, and therefore, ongoing and dynamic training updates will be an essential element of an effective CHW programme. This includes adding new evidence-based interventions and approaches in response to population health needs Public health emergencies, such as the COVID-19 pandemic, bring further and urgent attention to the need for ongoing and responsive training of CHWs CHWs are increasingly recognized as key contributors to and components of strong primary healthcare systems; training is a key prerequisite and incentive for CHWs to become and remain effective. Therefore, ensuring that training for CHWs is part of the larger and CHWs themselves are considered as part of the larger health workforce strategy and priorities is essential for achieving the Sustainable Development Goals and universal health coverage A strategy for the growth and development of CHWs is needed, so that they have an opportunity to develop themselves through career advancement opportunities	[6]
*Recent advances in supervision*	A review of the recent academic literature and grey literature on supervision of CHWs revealed that: Supervisors in CHW programmes are expected to perform a variety of roles covering administrative, clinical, and supportive activities Multiple supervision approaches are in use today: external supervision, community supervision, group supervision, peer supervision, and dedicated supervision Inadequate support for supervisors is a major challenge. With the right support, supervisors can help CHWs acquire and obtain other elements needed for strong performance, such as pay/incentives, role clarity, tools/supplies, and knowledge	CHW programmes now have the opportunity and the necessity of instituting stronger supervision as one of the critical steps in moving to a higher level of performance Increased, dedicated funding from national governments with support from the international community will be required to try new approaches to supervision, scale up existing approaches deemed promising, and evaluate the quality and effectiveness of such approaches alongside other health system improvement initiatives	[7]
*Motivation and remuneration*	The article reviews what kinds of incentives and remuneration have been offered to CHWs, how these incentives have influenced CHW motivation, and how programmatic and contextual factors have shaped the relationships between incentives and CHW motivation There is a wide variety of incentives offered in CHW programmes around the world, and these function at individual as well as health system and community levels CHW motivation can be affected by tensions between altruistic and material imperatives, by the social dimensions of their relationships to members of the health system and community, and by cultural, economic and political contexts CHW motivations are likely to change over time, as circumstances change, which means that CHW programmes must be able to assess and respond to changes in the effectiveness of various incentives WHO guidelines on CHWs have recently emphasized the importance of decent working conditions and fair labour practices in CHW programmes	Sustaining CHW motivation requires focused and consistent investment in locally meaningful and sustainable forms of incentives, typically with some clear support from or involvement from the state. CHW motivation cannot be sustained if CHWs are seen as a temporary solution to health system failures Sustaining CHW motivation requires thinking about incentives as multidimensional and about CHW motivation as something that changes over time All of this in turn requires a health system that is functional and effective enough to provide a clear role and proper support for CHWs	[8]
*Relationships with communities and health systems*	A major challenge of large-scale CHW programmes is that CHWs need to establish and maintain constructive relationships with the actors in the national health system as well as with actors in the community, and they need to navigate these dynamic relationships over time For CHWs to be optimally effective, they need to be embedded in the community as members who are well known, trusted and appreciated by community leaders, community members, and influential local groups	CHWs need a clearly defined role that is well understood and respected, and they need to be provided with functional supports from the health system and from the community Community engagement in CHW programmes is a process that requires leadership at all levels in the CHW programme as well as support from the health sector, local government, and community organizations	[9]
*Performance assessment*	A variety of existing frameworks is useful when assessing the performance of CHW programmes CHW programme performance assessments need to consider measurement of individual CHW performance and community-level outcomes, as well as to look at health systems and other contextual factors that influence CHW programme performance	CHW programme assessments ideally need to be based on data derived from a mix of reliable sources and by using a mix of methods to obtain these data Investment in CHW programme performance assessments is instrumental for continually innovating, upgrading, and improving CHW programmes at scale	[10]

In this final paper, we seek to identify opportunities for building stronger CHW programmes and greater community engagement. We see this as a means for achieving global health goals and strengthening health systems throughout the world to reach their full potential and to achieve “Health for All”, where health inequities both within and between countries are minimal, as envisioned at Alma-Ata more than four decades ago.

In 2018 the world celebrated the 40th anniversary of the 1978 Declaration of Alma-Ata and reaffirmed its basic principles, calling for a renewed global commitment to the goal of universal health coverage (UHC) through primary healthcare (PHC) as defined in the original Declaration [14]. In 2019, the United Nations General Assembly unanimously passed a resolution [15] stating that “measurable acceleration is urgently needed” to reach the health-related targets of the Sustainable Development Goals (SDGs) by 2030—namely UHC and ending preventable child and maternal deaths. Unless progress toward this goal accelerates, up to one third of the world’s population will still remain underserved by 2030 [15].

Not since Halfdan Mahler was Director-General of WHO from 1973 to 1988, has WHO had a leader as passionate about PHC and community health. Dr. Tedros Adhanom led the transformation of Ethiopia’s health system from a focus on hospitals and urban populations to an emphasis on strong community-based PHC programmes in which vertical programmes were effectively delivered at the community level through a common PHC platform. As a result, Ethiopia became a global leader in attaining the Millennium Development Goals for maternal and child health, HIV/AIDS, malaria, and tuberculosis [16, 17] and in accelerating progress in achieving UHC [17].

Tedros Adhanom’s leadership is now helping to guide WHO toward embracing more fully the goal of stronger PHC and community health programmes. And, for him, the platform of community-based service delivery by CHWs and engagement of communities as partners and resources are fundamental to the concept of PHC and community health [18]. As he said in his address to the World Health Assembly in 2019, “[T]here will be no UHC without PHC” [18]. Similarly, it is fair to say, particularly in resource-constrained, high-mortality settings, there can be no PHC without CHWs. CHWs need to be understood as the foundation of PHC as well as an increasingly important component of health systems in settings where delivery or equity gaps are present. The 2019 World Health Assembly resolution highlighted the importance of CHWs for achieving global public health goals [19] and called for the adoption of recently released WHO guidelines on health policy and system support to optimize CHW programmes [12].

CHW programmes are at a historical moment in which their contribution to health systems and population health improvement is increasingly being recognized as an integral part of health systems, particularly (but not exclusively) in low- and middle-income countries (LMICs), rather than continuing to be an underfunded afterthought. The growing recognition of the value of CHW programmes reflects the following key factors:

1. Evidence of the effectiveness of adequately trained and supported CHWs in providing quality health services and improving population health continues to grow [13, 20–23]. This evidence indicates that the number of lives of mothers and their children that can be saved each year by increasing the population coverage of the evidence-based promotive, preventive, and curative interventions provided by CHWs is even greater than the number that can be saved by expanding facility-based services at PHC centres and hospitals (1.7 million versus 2.3 million) [21, 24], although of course both strategies must be pursued in tandem.
2. Countries that have been making the strongest gains in population health outcomes have strong CHW programmes; Bangladesh, Brazil, Ethiopia, Nepal, and Rwanda are prominent examples [25].
3. WHO has, for the first time, created a set of guidelines for optimizing the contribution of CHW programmes to health systems [12].

The COVID-19 pandemic has made very clear the need for health workers on the ground who can visit homes to provide education about COVID-19, assist in identifying cases and tracing contacts, linking cases to treatment, and supporting vaccination [26]. The result is that the pandemic has led to calls throughout the world for CHWs to receive the proper respect and status that they deserve, not to mention the personal protective equipment they need to safely carry out their duties [27]. For example, the pandemic has led to a call for the creation of a CHW cadre in the United Kingdom that would continue, for the foreseeable future, to link all households with a CHW who can assist in addressing health needs in the household [28]. Similar proposals are emerging in the United States [29, 30].

On a broader scale, we are in the midst of a process some are calling “the third global health transition” [31] or “a grand convergence” [32]—emerging from a demographic transition (from higher to lower death and birth rates) into an epidemiologic transition (from a high burden of maternal and child conditions and communicable diseases to noncommunicable diseases [NCDs]). The world’s sights are shifting to achieving UHC, ending preventable child and maternal deaths, ending HIV/AIDS, controlling malaria and tuberculosis, controlling NCDs, and, most recently, to anticipating and responding to rapidly progressing epidemics and pandemics, which threaten health systems themselves. As the world’s wealth, technology, knowledge, and skills increase, persistent health disparities and untimely deaths from readily preventable or treatable conditions become even more unjust and morally unacceptable. There are unprecedented new technologies, tools, and programmatic approaches which offer the prospect of increasing the effectiveness of CHWs, provided they are giving appropriate training and support.

## Current challenges facing national CHW programmes

Key message box 2 In a recently produced compendium of case studies of national CHW programmes, the most commonly cited challenges were (1) inadequate programme financing, (2) interruptions in the supply chain for basic supplies, commodities, and medicines, (3) inadequate remuneration of CHWs, and (4) inadequate or poor-quality supervision.

A recently published set of case studies [13] provides insights into the structures, achievements, and challenges faced by national CHW programmes (Box 1). In each of the case studies, the authors describe the major challenges that these programmes face. The findings, summarized in Table 2, provide a realistic, grounded view of the constraints under which such programmes are currently functioning. Each national CHW programme faces many challenges of all sorts. What Table 2 provides is a listing of those that were considered by the authors of the case studies to be the most pressing challenges for that programme. Therefore, it does not provide an all-encompassing list of all the challenges these programmes face. The four most frequently seen challenges concern the supply chain for key programme commodities used by CHWs, programme financing, CHW compensation, and supervision. Each of these challenges was found to be a prominent issue for over half of the programmes documented in this set of case studies.

**Table 2:** Challenges cited by authors of 29 case studies of national CHW programmes [13]

Type of challenge	Number of national programmes for which this challenge was mentioned	Countries in which they challenge was mentioned and name of CHW cadre involved
Lack of supplies and commodities	20	Ethiopia (HEW, WDA); India (ANM, AWW, ASHA, VHG); Kenya (CHW); Liberia (CHA, CHSS); Malawi (CHW); Mozambique (APE); Niger (ASC, RV); Rwanda (*Binome*, ASM); Sierra Leone (CHW); South Africa (WBPHCOT); Zambia (CHA, CBV); Zimbabwe (VHW)
Inadequate, unstable, or unsustainable financing	20	Brazil (ASC Agent); Ghana (CHO, CHV); Indonesia (*Kader*); Liberia (CHA, CHSS); Malawi (CHW); Mozambique (APE); Myanmar (AMW, CHW, MV, TBV, TBA), Pakistan (LHW); Rwanda (*Binome*, ASM); Sierra Leone (CHW); South Africa (WBPHCOT); Tanzania (CHW); Zimbabwe (VHW)
Low compensation of CHWs	17	Brazil (CHW); Ghana (CHO, CHV); India (ANM, AWW, ASHA, VHG); Iran (B, MS), Kenya (CHV); Malawi (CHW); Mozambique (APE); Pakistan (LHW); South Africa (WBPHCOT), Zambia (CHW, CBV); Zimbabwe ( VHW)
Inadequate supervision	15	Brazil (ASC-agent); Ethiopia (HEW, WDA); India (ANM, AWW, SAHA, VHG); Mozambique (APE); Niger (ASC, RV); Rwanda (*Binome*; ASM); South Africa (WBPHCOT); Zambia (CH-assistant, CBV)
Management challenges	11	Brazil (ASC-agent); Ghana (CHO, CHV). Iran (B, MS); Kenya (CHV); Malawi (CHW); Nigeria (CHW, CHIPS, CDD); Tanzania (CHW)
Overall shortage of human resources	10	Indonesia (Kader); Liberia (CHA, CHSS); Kenya (CHV), Madagascar (AC, ACN); Malawi (CHW); Niger (ASC, RV), Pakistan (LHW)
Weak linkage with the formal health system	10	Brazil (ASC-agent); Ethiopia (HEW, WDA); Kenya (CHW), Malawi (CHW). Myanmar (AMW, CHW, MV, TBV, TBA)
Unavailability of health facilities	9	India (ANM, AWW, ASHA, VHG); Iran (B, MS); Malawi (CHW), Niger (ASC, RV)
Deficient training	8	Brazil (ASC-agent); Ethiopia (HEW, WDA); India (ANM, AWW, ASHA, VHG); Mozambique (APE)
Low level of community involvement	7	India (ANM, AWW, ASHA, VHG); Thailand (VHV); Kenya (CHV); Malawi (CHW)
Inappropriate workload	7	Ethiopia (HEW, WDA); Rwanda (*Binome*, ASM); Malawi (CHW); Zambia (CHA, CBV)
Role constraints	6	Nepal (FCHV); Nigeria (CHEW, CHIP, CDD, CHA); Zambia (CHA, CBV)
Limited career progression	6	Bangladesh (FWA, HA, CHCP); Ethiopia (HEW, WDA); Mozambique (APE)
Fragmented (vertical) programming	5	Myanmar (AMW, CHW, MV, TBV, TBA)
Low quality of services	5	Bangladesh (FWA, HA, CHCP), Kenya (CHV); Tanzania (CHW)
Violence and sexual harassment against CHWs	4	India (ANM, AWW, ASHA, VHG)
Increased health needs	4	Bangladesh (SS, SK); Madagascar (AC, ACN)
Discrimination	4	Bangladesh (SS, SK); Iran (B, MS)
Programme inefficiencies	4	Afghanistan (CHW); Ethiopia (HEW, WDA), Kenya (CHV)
Lack of political support for the CHW programme	3	Indonesia (*Kader*), Kenya (CHV); South Africa (WBPHCOT)
Limited educational background of CHWs	2	South Africa (WBPHCOT); Thailand (VHV)
High turnover of CHWs	2	Brazil (CH-agent); Kenya (CHV)

AWW: *aganwadi* worker; AC: *agent communautaire;* ACN: *agent communautaire nutritional*; AMW: auxiliary midwife; ANM: auxiliary nurse midwife; APE: *agent polivalente elementares*; ASC: *agent de santé communautaire;* ASHA: accredited social health activist; ASM: *agent de santé maternelle*; B: *behvarzs*; CBV: community-based volunteer; CDD: community-directed distributor; CHA: community health agent; CHCP: community healthcare provider; CHEW: community health extension worker; CHIP: community health influencer and promoter; CHO: community health officer; CHSS: community health service supervisor; CHW: community health worker; CHV: community health volunteer; FWA: family welfare assistant; FCHV: female community health volunteer; HA: health assistant; HEW: health extension worker; LHW: lady health worker; MS: *moraghebe-salamat*; MV: malaria volunteer; RV: *relais* volunteer; SK: *shasthya korbi;* SS: *shasthya shebika* TBA: traditional birth attendant; TBV: traditional birth volunteer; VHG: village health guide; VHV: village health volunteer; WBPHCOT: ward-based primary healthcare outreach team; WDA: Women’s Development Army; VHW: village health worker

Box 1. Contents of the book, *Health for the People: National Community Health Worker Program from Afghanistan to Zimbabwe* [13] The book contains 29 case studies of national CHW programmes from Afghanistan, Bangladesh, Brazil, Ethiopia, Ghana, Guatemala, India, Indonesia, Iran, Kenya, Liberia, Madagascar, Malawi, Mozambique, Myanmar, Nepal, Niger, Nigeria, Pakistan, Rwanda, Sierra Leone, South Africa, Tanzania, Thailand, Uganda, Zambia, and Zimbabwe. Each case study has at least one author who has personal in-country experience with the programme described, and each follows a common format to facilitate comparisons across cases. The studies look at a mix of CHW types, from the more professionalized end of the spectrum to less formalized volunteer CHW programmes. The programmes described are drawn from diverse regions and from both low-income and middle-income countries.

## How can national CHW programmes be strengthened?

Key message box 3 The strengthening of national CHW programmes will require building stronger leadership among those who understand the importance of CHW programmes and are passionate about strengthening these programmes. These people, in turn, can help to build national political support for expanding government support for these programmes. Increased donor support is needed, as are investments in rigorous programme monitoring, evaluation, and implementation research to enable national CHW programmes to reach their full potential.

Drawing on the papers in the series, we discuss below key questions related to strengthening CHW programmes and outline our thoughts on how these might be addressed. These suggestions are not intended to be definitive, and we would welcome further ideas and discussion. As we note below, innovations should be linked with ongoing monitoring and evaluation using appropriate methods.

### How can we foster leadership and passion for CHW programmes globally and locally?

- Amplify CHW voices by identifying and supporting CHWs to participate in meetings where they share their experiences. For example, the CHW Advocates campaign trains CHWs to demand their inclusion in high-level decision-making forums [33].
- Identify and support CHWs who are leaders, including CHWs who have become political leaders, so that CHWs can act as their own best advocates.^1^ For example the “120 under 40” programme recognizes and supports young leaders from the family planning movement [34].
- Identify young, unrecognized, potential leaders who already have a passion for CHWs and give them a platform to promote the CHW movement.
- Reach out to individuals and groups who are sceptical about or critical of CHWs, to engage them in discussion.
- Bridge the divide that often exists between preventive- and curative-oriented health workers.
- Emphasize the need for and uses of CHWs in high- and in middle-income countries as well as in low-income countries by showing how CHW programmes can address important health needs across settings.

### How can we mobilize greater investments in national CHW programmes and community platforms?

- Work with governments to develop long-term strategies for increasing sustainable, domestic support needed for strong CHW programmes (as the Financing Alliance for Health [35] is doing).
- Develop strategies to build political will that can lead to more domestic investment for national CHW programmes [36].
- Look for opportunities to cross-subsidize CHW programmes from other revenue streams within ministries of health, from the government more broadly, and from nongovernmental organizations (NGOs).
- Encourage greater technical support from international agencies.
- Increase financial support from donors for strengthening national integrated CHW programmes. Only 2.5% of global public health donor assistance over the past decade has been devoted specifically to CHW programmes, and two thirds of this was for vertical programmes related to HIV/AIDS, malaria, tuberculosis, reproductive health, or family planning [37].
- Encourage multilateral and bilateral organizations to assist governments in finding sustainable global and domestic resource streams that can be used for CHW remuneration.

### How can we reduce the gap between evidence and practice for increasing the population coverage of evidence-based interventions that CHWs can deliver (especially for reducing neonatal and child mortality)?

- Use evidence-informed implementation strategies [38] and consider well-described barriers and facilitators to implementing CHW programmes [39] and local evidence on factors affecting implementation and how these might be addressed.
- Further develop, expand, and evaluate (using appropriate designs) programmes building on robust evidence and experience at smaller scale, such as that for women volunteers who visit their neighbours frequently and who form Care Groups, used in 30 countries around the world [40–42], and for frequent home visits by CHWs in peri-urban Mali [43], Senegal [44], and elsewhere [45, 46].
- Explore whether dual-cadre CHW programmes (using minimally trained volunteer CHWs working with full-time salaried CHWs or paramedical workers) can make it possible to provide the frequent home visits needed to reduce neonatal and child mortality [47].
- Explore what can be learned, scaled up, and further evaluated from exemplary programmes, including the CHW programmes analysed by Exemplars in Global Health [25].

### How can CHWs contribute to stronger linkages at the local level for reproductive and maternal health?

- Develop stronger linkages for CHWs in the provision of family planning, maternal care, neonatal care, and other reproductive health services (such as mitigating gender-based violence and screening and treatment of cervical and breast cancer). For example, with women’s support groups engaging with CHWs, building on the evidence-based empowerment strategies of CHW-led participatory learning and action groups used successfully in Bangladesh, India, Malawi, and Nepal for reducing maternal and neonatal mortality [48, 49] and CHW-led Care Groups used by NGOs throughout the world [50].
- Develop CHW promotion of care-seeking at birthing facilities providing culturally appropriate, community-supported, high-quality care during delivery, building on experiences from rural Guatemala [51] and the urban low-income settlements of Bangladesh [52].

### How can we facilitate the expansion of CHW roles beyond their traditional maternal/reproductive, child health, and nutrition role without overloading them or diminishing their effectiveness?

- The Brazilian experience informs us that a comprehensive, life-course approach is entirely feasible providing that the number of households assigned to each CHW is reasonable (currently 150–200 in Brazil) [53].
- Based on the available evidence on which interventions for NCDs (including mental health conditions) can be effectively delivered by CHWs [54–59], consider whether incorporating some or all of these functions into existing CHW programmes is feasible, acceptable, promotes equity, and is cost-effective.
- Support research into how to sustain at scale the role of generalist CHWs (not funded under vertical programmes) in:
  1. identifying those with conditions of public health importance (e.g., HIV, tuberculosis, hypertension, diabetes, obesity, mental illness, persons with surgically treatable conditions) as well as conditions of local need (e.g., readily preventable or treatable health problems of the elderly such as presbyopia, palliative care for those with terminal conditions, pain control, basic needs for improved water and sanitation, and so forth),
  2. linking them to treatment, and
  3. serving as first-line providers of treatment under controlled conditions, with appropriate supervision and support.
- Support further research on the contributions of CHW programmes at scale in the detection and control of infectious disease outbreaks.
- Support case studies, field trials, and robust process and impact evaluations of programmes that incorporate new CHW roles.
- Support further research on the potential contributions of CHWs to vital events registration as well as to civil birth and death certification, and verbal autopsies.
- Engagement of local communities with the findings as well as field trials of these activities [60–63].
- Explore real-world implementation issues for CHW programmes with these expanded roles, determining what is necessary—in specific settings—for CHWs to perform these tasks effectively under routine programme conditions (including assessing the implications for the numbers of CHWs required to avoid overburden of tasks).

### How can vertical programmes (with their specific selective technical interventions) be better integrated within the national PHC system, including in national CHW programmes?

- Document examples of how complicated and unnecessarily difficult the training, implementation, and reporting of CHW work can be when different vertical programmes are engaging the same CHWs in an uncoordinated manner.
- Document current examples of how integration has been achieved [64, 65].
- Assess the implications for the numbers of CHWs for given catchment areas.
- Afghanistan’s, Ethiopia’s, and Rwanda’s approaches to these issues are exemplary [13].

### How can we support more information sharing, research, and evaluation of national CHW programmes?

- Support information hubs and communities of practice at the global and national levels, and CHWs’ access to these, including via online channels. The webstie CHW Central [66] is one example. Healthcare Information for All (HIFA), as another example, has established a working group for CHWs with the aim of promoting discussion on their health information and learning needs, and how these needs can be more effectively addressed [67].
- Provide opportunities for CHWs themselves to participate in decision-making structures as well as to express their views on their work and how they could become more effective. International forums generally have few, if any, CHWs in attendance. The efforts to engage CHWs at the first and second International Symposium on Community Health Workers (held in Kampala, Uganda, in 2017 and in Dhaka, Bangladesh, in 2019) are positive examples. The experiences this year with international conferences conducted entirely on a remote basis (due to COVID-19 restrictions) provide useful examples of how such convening can be done on a more inclusive basis than in the past.
- Build more communication capabilities for learning and sharing about CHW programmes and about CHWs themselves. Two current examples are (1) the Hesperian Health Guides programme [68], with its long experience in preparing and sharing materials for community health programming, and (2) the Last Mile Health’s Community Health Academy [69].
- Support research on and evaluation of all types of CHW programmes, but particularly national CHW programmes. Further research is needed on the effectiveness of national CHW programmes and community health platforms that focus on implementation issues in large-scale, real-world settings. Both independent research and evaluations paid for by the governments themselves are important. The recent evaluation of the CHW programme in Ethiopia is an important example [70].
- National CHW programmes that are seen as models deserve intense scrutiny to understand how they operate and what appear to be key factors contributing to their performance, so this information can guide policy and programming in other countries. This involves rigorous assessments of key components. New methodologies for this approach are emerging such as the multiphase optimization strategy (MOST), which is a new methodological approach for building, optimizing, and evaluating multicomponent interventions. Conceptually rooted in engineering, MOST emphasizes efficiency and careful management of resources to move intervention science forward steadily and incrementally. MOST can be used to guide the evaluation of research evidence, develop an optimal intervention (the best set of intervention components), and enhance the translation of research findings [71].
- Make better use of routine health management information system data for regular assessments and ongoing performance management of CHW programmes, particularly at the local level.

### How can we institutionalize the principles of mapping every household, visiting every house regularly, identifying local epidemiological priorities based on local data obtained from home visits, and working collaboratively with communities to address local health priorities?

- Develop further documentation on how programmes based on these principles have been implemented and assess their strengths and weaknesses.
- Assess the utility of the health information systems used in these programmes (family health folders linked to numbered households on village maps as has been implemented nationally in Ethiopia) and and how they can strengthen CHW programme effectiveness, including their linkage to vital events registration, cause of death analysis, and community engagement [72].
- Evaluate the added value of these approaches for promotion of equity.
- Document how CHWs, communities, and the peripheral health system can use these approaches to adapt national guidelines to the local context.
- Evaluate the utility of different health information systems used by CHW programmes, including how these strengthen CHW effectiveness [73].

### How can we develop stronger CHW programming in fragile states and in the context of humanitarian disasters and epidemics/pandemics?

We know that fragile states and areas with armed conflict have higher levels of collateral mortality (especially for mothers and children) [74, 75] and that with adequate support, CHW programming is possible even when health facility functioning is compromised. We also know that CHWs can have important impacts in the midst of humanitarian disasters [76, 77]. Our new reality now appears to be one in which the COVID-19 pandemic will likely be here for several years (just as the 1918 flu pandemic). There will be future pandemics, and with climate change there are likely to be more extreme weather events leading to more humanitarian disasters [78]. Therefore, there is a need to:

- Explore ways to tap rapidly into existing latent capacity among community members with previous experience in programme implementation.
- Develop and implement strategies for rapid training and scaling up of CHWs in these settings.
- Strengthen the role of CHWs for surveillance and vital events monitoring at all times.
- Undertake research on the role of CHWs during pandemics such as COVID-19, including in relation to surveillance, community mobilization, contact tracing, and so forth [79]. The CORE Group Polio Project has had extensive experience using CHWs for these types of activities as they pertain to global polio eradication [80–83]. As Ballard et al. [26] concluded, in their recent overview of the importance of CHWs for responding to the COVID-19 pandemic globally:

The investments in the supply chain, compensation, dedicated supervision, continuous training, and performance management necessary for rapid community response in a pandemic are the same as those required to achieve universal health coverage and prevent the next epidemic. Strengthening high-quality healthcare delivery systems will save lives, not just during COVID-19, but always. [26]

### How can we strengthen the role of CHWs in under-resourced urban settings (including informal settlements in LMICs)?

- Gather evidence on the use of CHWs in informal urban settlements, where challenges regarding water, sanitation, hygiene, food safety, vector control, and waste management have to be dealt with in addition to other basic healthcare issues, especially for mothers and children, building on recent pioneering work [84, 85].
- Explore new approaches that are currently being used to strengthen health programmes in informal urban settlements using CHWs [86].

### What are the most appropriate roles for NGOs in CHW programming?

- NGOs have been global leaders in pioneering the development and implementation of programmes, in community engagement, and in the research and development components of community-based PHC [87, 88]. However, NGOs and their donors have sometimes undercut government leadership, pressured governments to pursue fragmented, disease-specific projects, and have instituted approaches that have been damaging to national health systems in the longer term. We need to explore ways of building on the strengths of NGO initiatives while also strengthening integrated, national health systems. Guiding principles for such engagement should be drawn on more widely [89].
- NGOs sometimes operate their own CHW programmes separate from government CHW programmes, while others are working hard to assist the government in strengthening government CHW programmes [89].
- NGOs have been global leaders in pioneering the development and implementation of CHW programmes as well as in community engagement [87, 88].
- In Afghanistan and South Sudan, among other countries, the government contracts with NGOs to run its national CHW programme. This is a model that deserves consideration for application in other settings, particularly where government services are weak and underdeveloped.
- Given the lack of authority that ministries of health in LMICs have in many urban areas (where municipal governments or national ministries of urban affairs have jurisdiction), the lack of government-run CHW programmes there, and the prominent role of the private sector in urban healthcare, NGOs can play an important role in CHW programming in urban areas as well as in implementation research pertaining to these programmes.
- NGOs have been global leaders in community health research and innovative program development [90]. They need to capitalize on this strength to continue to contribute to implementation research.
- While many policy makers consider NGOs as *boutique* operators that have less to offer solving global health problems at scale, there are important exceptions to this. BRAC’s^2^ CHW health programmes in Bangladesh reach over 100 million people and another 20 million people in other countries [88]. The CORE Group Polio Project coordinated the efforts of international and local national NGOs to engage communities and deploy CHWs at scale in 11 countries to address urgent needs in eradicating polio, strengthening immunization programmes more broadly, and responding to community priorities for maternal and child health [80, 83]. Moreover, many NGOs play important roles in advocating for and supporting changes in national policy—for instance, the recent provision of national-level support from multiple NGOs for community health strategy updates in Kenya.

### What are priority areas for CHW research and programme performance assessment?

- Follow the recommendations of the 2018 WHO guidelines and focus research on (1) CHW selection, education, and certification, (2) management and supervision, and (3) integration into and support by health systems and communities [12].
- Support operations research on how to (1) ensure CHWs are provided with more reliable supplies of needed programme commodities, (2) optimize CHW work time and task allocation [91, 92], and (3) balance CHW roles and responsibilities to households, community groups, and patients coming to them for curative care. At present, Ethiopia and BRAC have established exemplary programmes that address these issues in their programmes, and much can be learned by a closer analysis of how their programmes work [13, 25, 93].
- Support research on amplifying CHW leadership and voice, including around CHW labour rights, peer-to-peer engagement, and participation in policy decision-making.
- Support operations research on how to better provide both social and supervisory support for CHWs, notably when traditional approaches to supervision have failed or are impractical. Again, Ethiopia and BRAC are leaders.
- Investigate task overload, burnout, and career (de)motivation for CHWs. Ethiopia and Brazil are struggling with these issues and provide a good example of how these issues might be addressed.
- Develop better evidence on the full costs of strong CHW programmes and the comparative value of investing in them along with improved facility-based services as against the alternative (i.e., improving facility-based services without improved CHW services).
- Develop better documentation of how national CHW programmes function. *Health for the People* [13] is one small step in that direction.
- Build the evidence base on the role of CHWs in urban settings. Research in the area is quite limited at present.
- Continue to explore how we can use digital technologies to support CHWs in their work [72, 94].
- Build leadership and momentum for longitudinal evaluation and learning for adaptive community health platforms in partnership with communities, CHWs, and the local health system) [95].
- Further document and promote efforts to promote local government engagement for strengthening CHW programming [96, 97].
- Further document and promote community engagement strategies for the selection and priority setting of community activities, support to community-based structures, and involvement of community representatives in decision-making, problem-solving, planning, and budgeting processes as they relate to CHW activities [98].

## Discussion

Key message box 4 The global experience and the scientific evidence are unequivocal: CHWs are among the most cost-effective and the most rapidly implementable approaches to improve the health of underserved populations. Our task now is to provide national CHW programmes with the resources and technical support to enable them to reach their full potential.

As national CHW programmes grow increasingly important for health systems and community health, there will be a need for new thinking and, importantly, for stronger political and financial support. We also need to consider how to increase the population coverage of evidence-based interventions that CHWs can provide, how to adapt programme elements that have been used by other effective programmes, and how to redirect attention to epidemiological priorities. Mechanisms for strengthening the monitoring and evaluation of programmes, including expanded implementation research, are also critical.

### Change our mental models, giving greater recognition to CHWs and communities as central components of health systems

We are long past due for a shift in our understanding of CHW programmes, from viewing them as a temporary and underfunded afterthought, to seeing them as an integral component of optimally functioning health systems anywhere in the world, including high-income countries and LMICs.

At the same time, we need also to recognize communities as an integral part of health systems—a key component of the health system that was initially ignored when the systems *building blocks* were first conceptualized by WHO in 2007 [99]. The lack of inclusion of communities and community-based services in frameworks for health systems strengthening is striking [100]. An expansion of the six WHO building blocks has been proposed that highlights community-based service delivery as well as community mobilizing and organization, with attention to nontraditional health system components such as social capital, inter-sectoral partnerships, local governance, equitable financing, community information and data systems, and household production of health [101]. After all, as the most important stakeholders in the health system, individuals, households, and communities must be enabled to demand healthcare that is responsive to their needs and concerns, and that works collaboratively with them to improve their own health and well-being.

In recognizing that communities are integral to health systems, we do not mean to imply that facility-based services are not vital and should not also be strengthened. CHW programmes rely on support from their nearest facilities, and without adequately functioning facilities, patients referred by CHWs will not be able to receive proper care, thereby undermining CHW programme functioning.

CHWs are firmly ingrained in the mental models of many of us as being relevant only for poorer countries. We need to change this [102]. The number of CHWs in the United States is growing rapidly in response to persistent gaps in the health system [103, 104], and they are serving an important role in the COVID-19 pandemic response [29]. The United States Government’s Fiscal Year 2021 Reconciliation Act (American Rescue Plan) provides $7.6 billion in funding to public health departments to hire 100,000 full-time employees into the public health workforce. This will include CHWs, among others, who will hopefully become a permanent cadre in the health sector [105, 106]. As noted above, proposals have also been made to establish a permanent CHW cadre in the United Kingdom [28].

#### Provide more support for implementation research on CHW programming

In its guidelines for health policy and system support to optimize CHW programmes, WHO noted that although there is a rich body of literature on CHW programming, there is a striking lack of rigorous research on CHW programme effectiveness that can provide practical guidance on programme implementation [12]. Large-scale randomized controlled trials cannot provide the needed evidence. Evidence needs to come from comprehensive, critical programme case studies and from investigations not only on what works but also on what conditions are required for such programmes to perform well (including health system requirements) [13]. Given the great deal of evidence on the effectiveness of CHW programmes operating under ideal, small-scale and short-term conditions [20, 22], more research is needed on programmes operating at scale uunder routine conditions for the longer term. Building and making use of local capacity for this type of research is needed.

As mentioned above, the WHO guidelines draw attention to the need for research on selection and training, management and supervision, and integration into and support by health systems and communities [12]. Other work has identified further priority research questions including on effective policy, financing, governance, supervision and monitoring systems, the role of digital technologies, CHW preferences, drivers of CHW motivation and retention over time, and CHW rights and well-being [107].

A key need identified within this research agenda is to document and evaluate strategies that help to ensure that CHW programs work optimally, since there is already considerable evidence for effectiveness of the interventions they are expected to deliver. As Frankel observed almost three decades ago (in 1992),

There is no longer any place for discussion of *whether* CHWs can be key actors in achieving adequate health care. The question is *how* to achieve their potential. [108], p. 1

Thus, we need to determine what conditions need to be met at scale so that CHW programmes and community engagement can reach their full potential. For instance, Hazel et al. [109] have documented the disappointing results of evaluations of CHW programmes that employed integrated community case management (iCCM) for childhood illness in Burkina Faso, Ethiopia, and Malawi regarding the quality of care provided by CHWs, the low utilization of their services, the low population coverage of CHW services, and the absence of a demonstrated impact on mortality. This work concludes that instead of discarding iCCM as a component of CHW programming, we need instead to think of CHW programmes as requiring, just as human infants do, “steadfast attention, investment, and development guidance to mature and achieve their mortality reduction potential” [109]. Ongoing programme evaluations and implementation research are needed to continually refine, develop, and modify CHW programmes. And, importantly, these authors note that “[l]ocal capacity to collect and analyze relevant data is a prerequisite for generating essential knowledge and putting this knowledge to use” [109].

We also need to shorten the lag time between generation of evidence and its application. Too often we find ourselves faced with a dilemma—there is a lack of evidence to guide implementation, and there is a lack of implementation because of a lack of evidence. But there is also plenty of evidence that is not being acted upon.

#### Build stronger political and financial support

We need to build mutual support and collaboration among health workers, the lack of which has hindered effectiveness of CHW programmes. In many countries, professional associations of nurses and physicians have opposed the introduction of CHW programmes as well as efforts to delegate authority to CHWs to manage dispensing medication (e.g., childhood pneumonia and malaria). This has occurred even where we have robust evidence demonstrating that these tasks can be performed safely and effectively by well-trained and supervised CHWs [110]. We need to ensure that training encourages respect and complementarity among nurses, doctors, CHWs, and other health cadres rather than positioning one another as threats or opponents.

Further, we need to convince government leaders, political leaders, medical opinion leaders, civic society, and local grassroots leaders of the importance of CHW programmes, the value of strengthening them, and the benefits that can be achieved by expanding government financial support for them.

#### Expand the population coverage of evidence-based interventions

Based on modelling, it has been estimated that if we could raise the population coverage of current evidence-based maternal-child interventions that can be implemented at the community level by CHWs to 90% in the 74 countries with 97% of the child and maternal mortality in the world, an additional 2.6 million maternal and child deaths could be averted each year [111]. This does not include the additional benefits that would accrue by expanding community-based family planning services [111]. The interventions with the greatest potential still have low levels of population coverage.

#### Adapt programme elements that have been used by other effective programmes

A review of community-based PHC in improving maternal, neonatal, and child health [20] identified four strategies for which there is good evidence of effectiveness:

- home visitation,
- participatory women’s groups,
- community case management of childhood illness, and
- provision of outreach services by mobile health teams [112].

The evidence base for these strategies needs to be expanded further, and these strategies need to be more widely adopted and scaled up. Community case management of childhood illness, using proactive frequent home visitation, has shown promising results as a strategy for reducing under-five mortality in Mali and for improving access to care in Senegal, in which CHWs are well supplied with the medicines they need [43, 44]. Another promising approach is tiered CHW programme delivery: dual-cadre CHW programmes and even triple-cadre programmes are now emerging. Both approaches merit further replication and ongoing assessments.

#### Give continuing emphasis to the potential for CHWs to address the most frequent, serious, readily preventable or treatable conditions (i.e., epidemiological priorities)

This involves supporting CHW activities that directly address the leading causes of neonatal and perinatal mortality, childhood undernutrition, and under-five mortality (from pneumonia, diarrhoea, and malaria) [113]. It also involves identifying and linking pregnant women with appropriate care, identifying persons with unrecognized hypertension and diabetes, and providing them with first-line treatment or linking them to sources of facility-based PHC. Finally, it involves supporting those with long-term physical and mental disabilities.

#### Strengthen monitoring and surveillance

CHWs have the potential to identify local epidemiological priorities based on routine home visitation, including detection of disease outbreaks and registration of vital events (which can also be entered into the civil registration system), with support from mobile devices, as may be appropriate to the context [114–116]. These activities are particularly applicable in fragile and conflict-affected states where facility-based services are weak or nonfunctional.

## Conclusion

It is time to acknowledge the important contributions that CHW programmes can make. Programmes for CHWs and community health, integrated within national health systems, should share pride of place with other global health priorities and garner comparable levels of financial and policy support, technical leadership, and prominence in the organizational architecture of global health.

At the same time, it is necessary to recognize that CHW programmes and community health initiatives more broadly remain relatively underdeveloped areas of endeavour in global public health in terms of (1) the number of professionals working to advance the field, (2) investments in expanding the evidence base, and (3) investigations into the factors affecting implementation effectiveness at scale.

We now know enough to conclude that—when designed appropriately to context and adequately supported—CHW programmes and community health initiatives can accelerate progress in achieving population heath goals and in reducing health disparities. Support for new thinking, innovative programming, and research, including implementation research, is urgently needed, recognizing that a return on this investment of effort will take time.

Taking the longer-term view, now is the time to invest in the following:

1. Providing opportunities for political leaders, programme managers, and other stakeholders to visit exemplary national CHW programmes to learn, first-hand, how they have embedded CHW programmes in the community and integrated them within the national health system.
2. Exposing such decision-makers to local and international conferences and seminars where learning from various countries on these programmes is shared.
3. Providing decision-makers as well as community members and leaders with high-quality information on why national CHW programmes are important, on the financial, social, and technical conditions for their success, and on the need to strengthen them.
4. Developing better metrics that can be widely shared with communities on levels of government funding currently going to hospitals, PHC facilities, and to community outreach/CHW programming.
5. Assessing the past several decades of experience with the promotion of family planning, immunizations, and other vertical programmes, and incorporating lessons learned from strengthening these programmes into a strategy for strengthening PHC, including national CHW programmes.

In 2019, the United Nations called on all governments to invest an additional 1% of their GDP in expanding and strengthening their PHC systems in order to achieve UHC [117]. UHC will be required to achieve the SDGs. In order to make this additional investment optimally productive, a major share of this investment will need to be directed towards community initiatives, including CHW programmes. The current level of investment in CHW programmes and community health is poorly documented, but it will need to grow substantially [118].

A strong commitment by governments to community health initiatives, including CHW programming, will give a major boot at this critical moment will encourage donors and technical support organizations to follow suit. As Sherry, Ghaffar, and Bishai astutely observe, “without initiatives to help community health platforms flourish around the world, the health gains promised by [specific evidence-based technical] interventions will cost more and deliver less…. With the availability of local data, local forums for sharing data, and local multi-sectoral stakeholder engagement, [such technical] solutions will work better and deliver more” [119].

One of us (Mushtaque Chowdhury) recently noted:

In the long-term interests of the health care sector of the country, we believe CHWs should be systematically trained and integrated within the health system. We would like to see that they are recognized, at long last, as valued health professionals, something they are, and are paid as such too. This, for Bangladesh, is well within the realm of what is possible. Such choices should not be viewed as second-best solutions for the poor but the best bet for health, and for addressing this pandemic and others which are likely to come. We urge that we build on these tested local strengths as a priority rather than aiming to replicate high-cost medical interventions with limited, and in some instances, unproven, prospects of efficacy. [120]

Calls are being made for the establishment of a national CHW cadre in the United Kingdom [28] and for a community health corps in the United States [29, 30] to carry out similar functions—to assist in controlling the COVID-19 pandemic but also to strengthen the health system moving forward and to be able to be better prepared for future pandemics.

The potential for CHWs and for community engagement, more broadly, to serve as an integral component of PHC exceeds what can be achieved by facility-based care alone. This is true not only for ending preventable child and maternal deaths, but also for better controlling NCDs. Furthermore, in many countries, CHWs are the frontline of defence against the COVID-19 pandemic. Thus, there are now calls throughout the world for CHWs to be appropriately supported (and protected) to enable them to play a key frontline role against the pandemic and then, moving forward, a key part of the foundation of health systems.

By enabling national CHW programmes to reach their full potential, they in turn can lead the way toward accelerating progress in reducing inequities in health and achieving health for all. “If it happens in the community, it happens in the nation. It if does not happen in the community, it does not happen at all” (Miriam K. Were, personal communication).

## Data Availability

Any articles and other materials cited by the authors are available from the corresponding author.

## Abbreviations

AIDS: Acquired immunodeficiency syndrome
CHW: Community health worker
COVID: Coronavirus disease
HIV: Human immunodeficiency virus
iCCM: Integrated community case management (of childhood illness)
MOST: Multiphase optimization strategy
NCDs: Noncommunicable diseases
NGO: Nongovernmental organization
PHC: Primary healthcare
SDGs: Sustainable Development Goals
UHC: Universal Health Coverage

## Footnotes

1 There is a growing number of people, especially women, who had previously been CHWs who are now political leaders and elected officials.

2 BRAC (http://www.brac.net/) is the largest NGO in the world. The acronym originally referred to the Bangladesh Rural Advancement Committee and now refers to Building Resources across Communities.
